# Supplementary material for: Yiai Fuzheng decoction inhibits triple-negative breast cancer by remodeling the immune microenvironment
Source: Front Immunol. 2025 Sep 30;16:1615631. doi: 10.3389/fimmu.2025.1615631 (PMC12518410; doi:10.3389/fimmu.2025.1615631)
Supplement: Supplementary file 2 [file DataSheet1.zip › Supplementary File 2/Treatment/C_vs_H/04_OPLS_DA/Model_Validation/OPLSDA_Permutation.pdf]

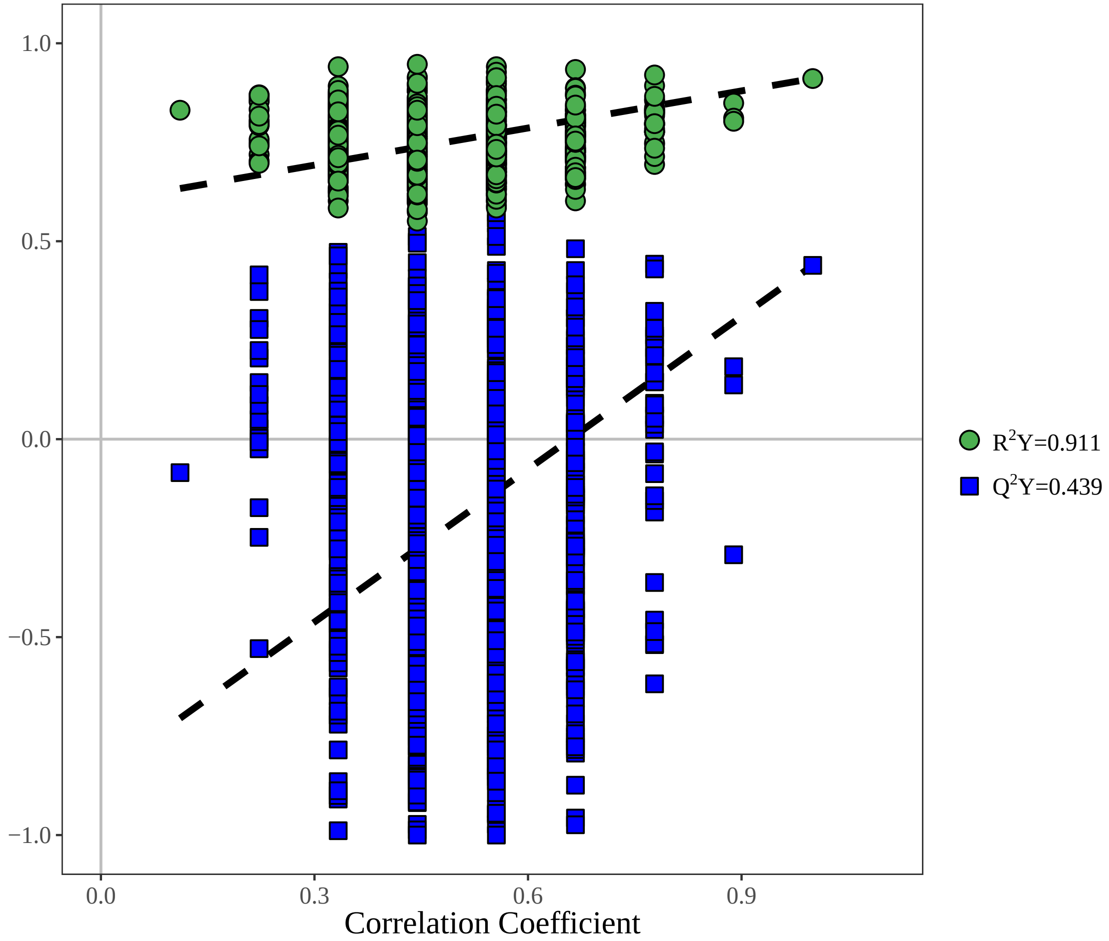
1.0
0.5
0.0
−0.5
−1.0
0.0
0.3
0.6
0.9
Correlation Coefficient
R2Y=0.911
Q2Y=0.439
